# Seeing the forest through the trees: improving decision making on the Iowa gambling task by shifting focus from short- to long-term outcomes

**DOI:** 10.3389/fpsyg.2013.00773

**Published:** 2013-10-18

**Authors:** Melissa T. Buelow, Bradley M. Okdie, Amber L. Blaine

**Affiliations:** Department of Psychology, The Ohio State University NewarkNewark, OH, USA

**Keywords:** decision making, Iowa gambling task, delay discounting, mood state, learning

## Abstract

**Introduction:** The present study sought to examine two methods by which to improve decision making on the Iowa Gambling Task (IGT): inducing a negative mood and providing additional learning trials.

**Method:** In the first study, 194 undergraduate students [74 male; *M*_age_ = 19.44 (*SD* = 3.69)] were randomly assigned to view a series of pictures to induce a positive, negative, or neutral mood immediately prior to the IGT. In the second study, 276 undergraduate students [111 male; *M*_age_ = 19.18 (*SD* = 2.58)] completed a delay discounting task and back-to-back administrations of the IGT.

**Results:** Participants in an induced negative mood selected more from Deck C during the final trials than those in an induced positive mood. Providing additional learning trials resulted in better decision making: participants shifted their focus from the frequency of immediate gains/losses (i.e., a preference for Decks B and D) to long-term outcomes (i.e., a preference for Deck D). In addition, disadvantageous decision making on the additional learning trials was associated with larger delay discounting (i.e., a preference for more immediate but smaller rewards).

**Conclusions:** The present results indicate that decision making is affected by negative mood state, and that decision making can be improved by increasing the number of learning trials. In addition, the current results provide evidence of a relationship between performance on the IGT and on a separate measure of decision making, the delay discounting task. Moreover, the present results indicate that improved decision making on the IGT can be attributed to shifting focus toward long-term outcomes, as evidenced by increased selections from advantageous decks as well as correlations between the IGT and delay discounting task. Implications for the assessment of decision making using the IGT are discussed.

## Introduction

Individuals are called upon to make decisions on a daily basis. Some of these decisions can be made through a calculated analysis of available options—weighing individual risks and benefits. This process has been termed “cold” decision making (Shafir et al., 1993; Seguin et al., 2007). Conversely, some decisions require individuals to rely instead on gut feelings and instincts. Decision making that involves this affective component is termed “hot” decision making (Damasio, 1994; Seguin et al., 2007). As many decisions can have long-term effects on an individual (Bechara et al., 1994; Denburg et al., 2006), it is important to understand not just how individuals make decisions, but whether the decision making process can be improved.

**Figure 1:**
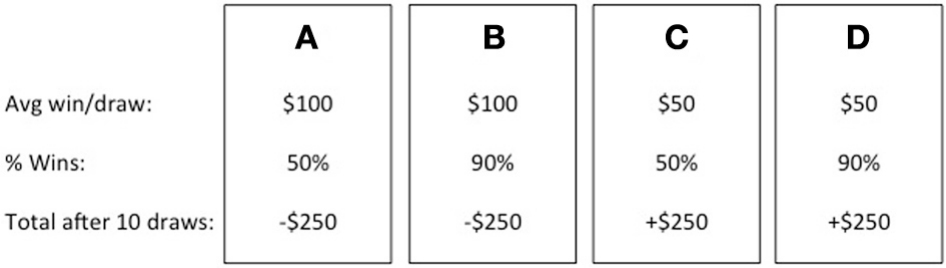
**Deck contingencies on the Iowa gambling task**.

One of the most popular behavioral measures of affective decision making processes is the Iowa Gambling Task (IGT; Bechara et al., 1994). The IGT was created to assess decision making impairments among individuals with documented damage to the ventromedial prefrontal cortex (VMPFC) who were experiencing real-world decision making deficits but performed within normal limits on formal assessment of executive functions. During the task, participants are given 100 trials in which to maximize profit. Selections are made from one of four decks of cards (A, B, C, D). On each selection, participants win money but also sometimes lose money (see Figure 1; Bechara et al., 1994; Bechara, 2008). Decks A and B provide an average immediate gain of $100, whereas Decks C and D provide $50. But, after 10 selections from Decks A or B, participants have incurred a net loss of $250. Ten selections from Decks C or D instead result in a net gain of $250. Based on these long-term outcomes, Decks A and B have been termed “disadvantageous” and Decks C and D “advantageous” (Bechara et al., 1994; Bechara, 2008). Performance on the IGT is typically broken down into 20-card blocks of trials; however, at the start of the task, participants are not told much about the relative risks and benefits of each deck. Thus, the first 40 trials have been termed decision making under ambiguity (Brand et al., 2007). The final 60 trials, in which the relative risks and benefits of each deck are better known, are instead termed decision making under risk. Disadvantageous decision making on this task is typically defined as continued selections from the disadvantageous decks during these later trials, and has been shown across multiple clinical samples (see Buelow and Suhr, 2009).

However, examining performance in this manner can mask differences in preferences for individual decks. For example, multiple studies have shown a Deck B preference (e.g., Toplak et al., 2005; Caroselli et al., 2006; Fernie and Tunney, 2006). Decks A and B differ in frequency and magnitude of immediate losses (Bechara, 2008). Deck A results in losses on 50% of trials, and the individual losses are smaller in magnitude. Deck B experiences much larger losses but on only 10% of trials. The same holds for Decks C (50% smaller losses) and D (10% larger losses). Thus, it is possible that differences in preferences between Decks A and B (and Decks C and D) can be attributed to individual differences in preference for a high frequency of positive short-term outcomes over long-term gains (Chiu et al., 2008). In real-world decisions, these facets are often juxtaposed. For example, cigarette smokers must weigh the immediate gains (removal of withdrawal symptoms) and long-term outcomes (health risks) in deciding to smoke each day. Alternatively, for individuals forming new romantic relationships in which the immediate gains and long-term outcomes are equally beneficial (i.e., both individuals elicit positive feelings in one another and have similar long-term goals), the focus on short- vs. long-term goals should not matter. However, if there is a discrepancy in these goals, then individuals would need to decide whether the short-term gains outweigh any long-term negative consequences.

The creators of the IGT put forth the somatic marker hypothesis to explain task performance. According to this hypothesis, the experience of emotion is critical to the decision-making process (Bechara, 2004). Gut feelings, or somatic markers, help guide decision making, even before individuals are consciously aware of this process. Multiple studies have shown the presence of anticipatory somatic responses before disadvantageous decisions (see Dunn et al., 2006, for a review). Processing of somatic markers by the VMPFC, amygdala, and other structures (Bechara et al., 1994, 1999; Davidson, 2002) could explain how individuals quickly learn to choose advantageously on the IGT. Thus, it is plausible that directly manipulating one's mood state may impact decision making processes. If emotions help guide decision making, deliberately putting someone in a positive or negative mood could alter their decision making.

Previous research has shown that subjective mood states can influence decision making processes (Schwarz and Clore, 1983; Forgas, 1995). Disadvantageous decision making on the IGT and other tasks is seen with self-reported negative mood state (Tice et al., 2001; Kaplan et al., 2006; Suhr and Tsanadis, 2007; Roiser et al., 2009a) however, others have shown more advantageous decision making with both negative (Smoski et al., 2008; Heilman et al., 2010) and positive (Nyren et al., 1996; Roiser et al., 2009b) mood state. Mood state can also affect the frequency of risk-taking behaviors (Johnson and Tversky, 1983; Arkes et al., 1988; Finucane et al., 2000). Being in a positive mood could help decision making by increasing creativity and problem solving (Isen, 1987; Murray et al., 1990; Gasper, 2004; Hirt et al., 2008), allowing for focus on available and general knowledge to arrive at a conclusion (Melton, 1995; Bless et al., 1996; Gasper and Clore, 2002; Ruder and Bless, 2003; Bramesfeld and Gasper, 2008). Positive mood could indicate a situation is benign, leading to approach-based behaviors (Gasper, 2003; Grawitch and Munz, 2005).

As the IGT was designed as a hot, affective decision making task, it is likely that mood state influences performance. Individuals in a negative mood may think through their selections more, leading them to prefer decks that maximize long-term outcomes. In fact, individuals who perform well on the IGT, in that they choose from decks with a long-term reward, engage in more effortful cognitive processes (assessed via a questionnaire regarding deck probabilities at task completion) compared to those with more disadvantageous performance (e.g., Busemeyer and Stout, 2002; Guillaume et al., 2009). Thus, negative mood may increase level of elaboration on each decision, in turn increasing cognitive deliberation on the task. This process may lead individuals to consider long-term instead of short-term outcomes, ultimately improving decisions.

Research that has directly manipulated mood has indicated both negative (Lerner et al., 2003; Yuen and Lee, 2003; Chou et al., 2007; Harlé and Sanfey, 2007; Cryder et al., 2008) and positive (de Vries et al., 2008) mood can lead to more advantageous decision making. When the IGT and a second behavioral decision making task, the Balloon Analogue Risk Task (BART; Lejuez et al., 2002) were examined, Heilman and colleagues (2010) found emotional regulation, not manipulation of negative mood, was the factor that affected decision making. Others have examined mood and financial decision making, showing negative mood increased impatience and lead to an inability to delay gratification for larger future rewards (Lerner et al., 2013). Of the literature that has manipulated mood state, few have used the IGT in particular (see de Vries et al., 2008; Heilman et al., 2010, for exceptions). Manipulating, rather than measuring, mood allows for a more stringent examination of the impact of mood on decision making.

Recent research has also begun to investigate a second means of potentially improving decision making—adding learning trials. Providing additional trials on the IGT reversed a decision making impairment among individuals with Parkinson's disease (Buelow et al., 2013) and in a small non-clinical sample (Lin et al., 2013). Studies of decision making using the IGT have shown that some individuals fail to develop a deck preference (e.g., Peters and Slovic, 2000; Mueller et al., 2010), so providing additional trials may result in more advantageous decision making for this subset of individuals. In Study 2, we sought to examine a largely unexamined means of improving decision making: providing additional learning trials. In addition, performance on the delay discounting task (Kirby et al., 1999) was assessed to determine if temporal focus could be seen across tasks.

Individuals are asked to make decisions in everyday life in which choices must be made between immediate and delayed rewards. In some cases, the immediate reward is of a smaller magnitude than the delayed reward, but the higher value of the delayed reward is offset by the length of time until its receipt (Kirby and Herrnstein, 1995). Although most individuals prefer an immediate reward, if given a choice (Green and Myerson, 2004), those who do choose the immediate reward can be viewed as impulsive (Rachlin and Green, 1972; Teuscher and Mitchell, 2011), whereas those who “hold out” for the delayed reward are seen as able to delay gratification or inhibit impulses (Rachlin and Green, 1972; Appelhans et al., 2011). Delay discounting occurs when individuals discount (or decrease) the value of a delayed reward in favor of a smaller but more immediate reward (Rachlin and Green, 1972; Ainslie, 1974; Bickel et al., 1999; Green and Myerson, 2004).

Delay discounting (a preference for smaller, immediate rewards over larger but distant rewards) is seen across multiple addictive and risky behaviors (for reviews, see de Wit, 2008; MacKillop et al., 2011). In addition, performance on delay discounting tasks has been associated with real-world outcomes such as involvement in financial and other risk-taking behaviors (Kirby et al., 2005; Bickel et al., 2010; Sun and Li, 2010). It is believed that discounting of future rewards aids in real-world decision making by helping individuals differentiate between options (Kahneman and Tversky, 1979).

Researchers have also investigated the neurobiological underpinnings of delay discounting and the evaluation of temporal delays in decision making. Evidence suggests that the same structures implicated in IGT performance (i.e., orbitofrontal cortex and amygdala) are activated during delay discounting tasks (Bechara et al., 1994, 1999; McClure et al., 2004; O'Doherty, 2004; Shamosh et al., 2008; Ballard and Knutson, 2009). However, few have directly investigated correlations between the IGT and delay discounting tasks. Xu et al. (2013) found no correlation between the IGT and delay discounting. In a separate study, Perales et al. (2009) administered both the IGT and a delay discounting task, but failed to report a correlation between the two tasks. However, Sellitto et al. (2010) found that damage to the orbitofrontal cortex (i.e., a structure implicated in decision making on the IGT; Bechara, 2008), increased preference for smaller, more immediate rewards over larger but distant rewards. Thus, it is possible that performance on the tasks is linked.

Mood has also been shown to affect delay discounting. Inducing a positive mood moderates the relationship between impulsiveness and delay discounting, in that impulsive individuals in a positive mood discount future values to a greater degree than those in a negative mood (Koff and Lucas, 2011). Positive mood also affects the relationship between extraversion and delay discounting, in that extraverted individuals in a positive mood prefer more immediate rewards than extraverted individuals not in a positive mood (Hirsh et al., 2010). Inducing a negative mood, on the other hand, decreases delay discounting in a control sample (Lawrence et al., 2010). Thus, it is clear that mood state impacts the degree to which individuals discount future outcomes in favor of smaller but more immediate rewards.

### Present studies

The present studies sought to examine two potential methods by which decision making could be improved: inducing a negative mood and providing additional learning trials. Research using both self-reported mood and mood inductions indicates that negative mood can improve decision making on the IGT and other tasks (e.g., Nyren et al., 1996; Smoski et al., 2008; Roiser et al., 2009b; Heilman et al., 2010), whereas research into providing additional trials on the IGT is newer (Buelow et al., 2013; Lin et al., 2013). In Study 1, participants were randomly assigned to receive a negative, positive, or neutral mood induction prior to completion of the IGT. It was hypothesized that, when mood is manipulated and measured temporally close to the decision making task, individuals in a negative mood would exhibit more advantageous decision making on the IGT. In Study 2, participants completed the delay discounting task (Kirby et al., 1999) and two back-to-back administration of the IGT (200 trials total). We hypothesized that performance on the IGT would become more advantageous with additional trials, and that performance on the delay discounting task would be correlated with performance on the IGT (i.e., individuals would focus on short-term or long-term goals on both tasks).

## Study 1

### Methods

#### Participants

Participants were 194 undergraduate students (74 male; *M*_age_ = 19.44, *SD*_age_ = 3.69; 72.5% Caucasian) enrolled in psychology courses at The Ohio State University Newark and who received course credit for their participation.

#### Measures

***Measure of mood state.*** The 20-item Positive and Negative Affect Schedule (PANAS) was used to assess self-reported negative (10 items) and positive (10 items) mood state (Watson et al., 1988). Participants were asked to respond to each item as to how they felt in the present moment. For each subscale, responses to the 10 items were averaged, with higher scores indicating greater levels of positive or negative mood. Internal consistency was moderately high for both subscales (α = 0.78–0.87). The PANAS has been used in several previous studies that manipulated mood (e.g., Heilman et al., 2010; Wendrich et al., 2010).

***Decision making measure.*** The standard computerized IGT was used to assess decision making (Bechara et al., 1994; Bechara, 2008). The percent of selections from each deck were calculated for the decision making under ambiguity (Trials 1–40) and decision making under risk trials separately (Trials 41–100; Brand et al., 2007). Previous research has shown the importance of assessing selections from each individual deck due to differences in the frequency and magnitude of immediate losses (see Steingroever et al., 2013, for a review).

#### Procedure

The study procedure was approved by the Institutional Review Board at The Ohio State University, and all participants provided written informed consent. Participants first completed an assessment of their current mood state with the PANAS, and then completed a randomly assigned mood manipulation: positive (*n* = 63), neutral (*n* = 63), or negative (*n* = 68). Participants viewed a series of pictures in their assigned mood condition that were taken from the International Affective Picture System (IAPS; Lang et al., 2008)^1^. Average valence for each set, on a scale from 1 (*negative*) to 9 (*positive*), was as follows: negative 2.67, neutral 5.28, and positive 7.13.

Each picture was presented on a computer screen for 6 s, with a blank screen appearing for 3 s between each picture. To encourage participants to pay careful attention to each picture, they were told their memory for the pictures would be tested at the end of the stimulus presentation. After administration of the 20 “learning” trials, participants viewed a set of 35 pictures (consistent with the assigned mood condition) and indicated whether or not the picture was in the learning trial. Twenty of the 35 pictures were the same pictures seen during the learning trials, and the remaining 15 pictures were comprised of additional positive, negative, or neutral stimuli from the IAPS^2^. After the mood induction, participants completed a second rating of their current mood with the PANAS immediately followed by the standard computerized IGT.

#### Data analysis

In order to determine changes in mood, One-Way ANOVAs were conducted on the positive and negative PANAS scores at Time 1 and Time 2. To determine whether the mood manipulation affected preferences for individual decks during decision making under risk, MANOVAs were conducted with mood group as the between-subjects variable and percent selections from the individual decks on Trials 1–40 or Trials 41–100 as the within group variables.

### Results and discussion

#### Manipulation check

No differences were found between groups in positive, *F*_(2, 187)_ = 0.609, *p* = 0.545; or negative, *F*_(2, 187)_ = 1.260, *p* = 0.286; mood prior to the mood induction (see Table 1 for all means and standard deviations). The Positive mood group reported a significant increase in positive mood from Time 1 to Time 2, F_(2, 189)_ = 4.269, *p* = 0.015, η^2^_p_ = 0.043, and the Negative mood group experienced a significant increase in negative mood from Time 1 to Time 2, F_(2, 189)_ = 11.670, *p* < 0.001, η^2^_p_ = 0.110. The neutral group remained unchanged.

**Table 1:** **Study 1 variables presented as mean (standard deviation)**.

**Variable**	**Neutral**	**Negative**	**Positive**
PANAS-P T1	2.79 (0.79)	2.90 (0.78)	2.94 (0.82)
PANAS-N T1	1.50 (0.50)	1.50 (0.50)	1.38 (0.40)
PANAS-P T2	2.74 (0.82)	2.66 (0.81)	3.06 (0.82)^a^
PANAS-N T2	1.40 (0.53)	1.71 (0.82)^b^	1.21 (0.31)
PANAS-P T2-T1	−0.07 (0.58)	−0.21 (0.54)	0.13 (0.67)^c^
PANAS-N T2-T1	−0.11 (0.31)	0.17 (0.75)^b^	−0.17 (0.32)
**IGT PERCENT DECK SELECTION**			
**Trials 1–40**			
A	22.17 (8.21)	20.87 (7.44)	21.83 (5.87)
B	32.58 (11.45)	32.23 (12.52)	31.79 (9.92)
C	21.08 (6.30)	22.84 (9.63)	22.04 (6.13)
D	24.13 (9.20)	24.05 (15.29)	24.33 (9.96)
**Trials 41–100**			
A	16.92 (7.79)	15.86 (7.61)	16.97 (10.99)
B	30.22 (16.19)	30.23 (15.12)	27.36 (13.99)
C	18.86 (9.42)	23.84 (12.59)^b^	19.53 (8.08)
D	34.00 (17.45)	30.05 (16.46)	36.08 (20.45)

a Positive > Neutral, Negative.

b Negative > Neutral, Positive.

c Positive > Negative.

PANAS, Positive and Negative Affect Schedule; Positive (P) or Negative (N) subscale, pre-mood induction (T1) or post-mood induction (T2); IGT, Iowa Gambling Task; average percentage selections by deck for Trials 41–100.

#### Decision making performance

A repeated-measures ANOVA was conducted, independent of group status, to determine deck preferences on each set of trials. During Trials 1–40, participants showed a preference for Deck B over the remaining decks, *F*_(3, 555)_ = 35.846, *p* < 0.001, η^2^_*p*_ = 0.162. On Trials 41–100, participants selected more from Decks B and D than Decks A and C, *F*_(3, 555)_ = 43.583, *p* < 0.001, η^2^_*p*_ = 0.191. A MANOVA on the decision making under ambiguity trials (Trials 1–40) indicated no significant differences in deck selections between mood groups, Wilks' Λ = 0.979, F_(8, 360)_ = 0.477, *p* = 0.872, η^2^_*p*_ = 0.010. Results of the MANOVA on the decision making under risk trials (Trials 41–100) indicated a significant difference between mood groups, Wilks' Λ = 0.916, F_(8, 360)_ = 2.010, *p* = 0.044, η^2^_*p*_ = 0.043. Individuals in the Negative mood group selected significantly more from Deck C during the later trials than individuals in the Neutral (*p* = 0.007) and Positive (*p* = 0.020) mood groups. No differences were found for Decks A, B, or D (*p*s > 0.223).

The results of Study 1 indicated that inducing a negative mood immediately prior to a decision making task can alter decision making. Independent of mood group, participants preferred Decks B and D—two decks with different long-term outcomes but the same low frequency of immediate losses. However, a preference for Deck C during the final trials was seen among individuals in the Negative mood condition compared to those in the Neutral or Positive mood conditions. Deck C is considered an advantageous deck, in that it results in long-term gains, but is associated with immediate losses on 50% of trials. The present findings suggest that individuals in a negative induced mood began to select more from Deck C during the decision making under risk trials. However, the real-world applicability of this strategy is uncertain, as the finding would suggest that individuals should deliberately put themselves in a bad mood prior to making important decisions. That said, negative moods naturally occur in the real world and can aid in decision making based on shifting focus to long-term outcomes. For example, frequent negative interactions with a romantic partner should direct focus toward long-term outcomes and motivate further thinking on one's situation, leading to a different decision than the experience of frequent positive interactions.

The specific mechanism by which participants began to change their decision making strategy is unclear. Per the somatic marker hypothesis, the experience of emotions helps guide decision making on this task (Bechara, 2004; Dunn et al., 2006; Werner et al., 2009). It is possible a negative mood made participants more attuned to long-term outcomes. However, we should have also seen greater Deck C selections during the decision making under ambiguity trials (when somatic markers are most influential; Bechara et al., 2005). Instead, this preference occurred only on Trials 41–100, when increased cognitive processing occurs (i.e., not just emotions guide decision making on these trials; Maia and McClelland, 2004; Brand et al., 2007; Guillaume et al., 2009).

It may then be possible that mood state affected cognitive processing on this task. Individuals in a negative mood may have engaged in increased conscious, deliberative thought before making selections from each deck. Other research (not utilizing the IGT) indicates that when individuals are allowed to think freely (i.e., not constrained by time or other factors), positive mood may decrease information processing compared to neutral or negative moods (Mackie and Worth, 1989, 1991; Schwarz, 1990). In addition, individuals in a negative mood focus more on specific details (Bless et al., 1996; Gasper, 2003), which could help them understand the nuances of a particular situation or decision better (Gasper, 2003). In addition, those in a negative mood state are more sensitive to signals of punishment (Fredrickson, 1998), which could allow for a change in decision making strategy to avoid punishments (Fredrickson, 1998; Grawitch and Munz, 2005).

While the somatic marker hypothesis may explain early decisions on the IGT, cognitive processes likely influence later decisions. It may be that inducing a negative mood moderates the relationship between deliberative thought processes and decision making on the IGT; however, in the present study deliberative thought was not directly assessed. Future research investigating the influence of mood state, deliberative thought processes, and the relationship between these two factors on decision making on the IGT should directly assess these constructs.

## Study 2

### Methods

#### Participants

Participants were 276 undergraduate students (111 males, *M*_age_ = 19.18, *SD*_age_ = 2.58; 75.7% Caucasian) enrolled in psychology courses at The Ohio State University Newark and who received course credit for their participation.

#### Measures

***Iowa gambling task.*** The standard computerized IGT was utilized (Bechara et al., 1994; Bechara, 2008); however, an additional 100 trials were administered. Performance was broken down by the percentage of selections from each individual deck on Trials 1–40 (Block 1), Trials 41–100 (Block 2), Trials 101–140 (Block 3), and Trials 141–200 (Block 4).

***Delay discounting task.*** The 27-item Kirby et al. (1999) delay discounting task was utilized. Participants chose between a series of small, immediate rewards and delayed rewards of increased size. For example, participants chose between receiving “$55 today or $75 in 61 days” (Kirby et al., 1999). Previous research using this task has shown individuals who prefer immediate rewards are more impulsive and engage in higher levels of drug-using behaviors (Madden et al., 1997; Kirby et al., 1999). In the present study, *k*-values indicating the extent of the delay discount were calculated, with higher values indicating a preference for immediate gain over larger but distant rewards.

#### Procedure

The study procedure was approved by the Institutional Review Board at The Ohio State University, and all participants provided written informed consent. Participants completed the delay discounting task and the standard computerized IGT (100 trials), and then the IGT was immediately restarted for an additional 100 trials. All card decks refilled prior to the start of the second 100 trials.

#### Data analysis

Repeated-measures ANOVAs were used to assess preferences for decks on each of the four blocks of trials. Thus, four repeated-measures ANOVAs were conducted with deck (A, B, C, D) serving as the sole within-subjects factor. Only significant *post-hoc* results are reported below. Of note, participants were forced to choose from one of the four decks on each trial. A selection from Deck B, for example, indicated that the individual could not have selected from the other decks (i.e., selections are linked on each trial). Correlations were also calculated between *k*-values on the delay discounting task and IGT Block 2 and Block 4 performance for Decks A, B, C, and D separately. Due to study time constraints, valid data on the delay discounting task was available for only 180/276 participants.

Additional analyses were conducted to further examine the preference for short- vs. long-term rewards and the relationship between delay discounting and IGT performance. Previous research has compared the preference for Deck B over Deck D (Buelow and Suhr, in press), as these decks have the same frequency of losses (10% of trials) but different long-term outcomes (deficit with B and gain with D). Ratios of selections from Deck B divided by selections from Deck D (B/D) were calculated for Block 2 and Block 4. Larger B/D ratios indicate a relative preference for Deck B over Deck D, and smaller B/D ratios indicate a relative preference for Deck D over Deck B.

### Results and discussion

#### IGT analyses

**Figure 2:**
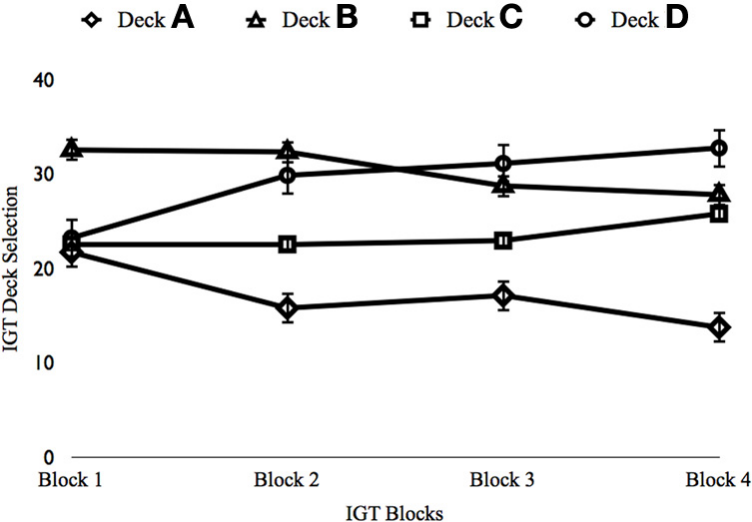
**Depiction of IGT Performance in Study 2**. *x*-axis, IGT Block; *y*-axis, Percent selections from each deck. Error bars reflect standard errors.

Results of the ANOVA on Block 1 indicated a significant effect, F_(3, 822)_ = 59.680, *p* < 0.001, η^2^_*p*_ = 0.179 (see Figure 2). Participants showed a preference for Deck B over the remaining decks (*p*s < 0.001). Significant differences in deck selections were also seen during Block 2, F_(3, 822)_ = 54.856, *p* < 0.001, η^2^_*p*_ = 0.167. Participants favored Decks B, C, and D over Deck A (*p*s < 0.001), the deck associated with “pathological decision making” (Bechara, 2008).

Next, the additional 100 trials were examined. A significant effect was found for Block 3, F_(3, 822)_ = 33.478, *p* < 0.001, η^2^_*p*_ = 0.109. Participants again selected more from Decks B (*p*s < 0.001) and D (*p*s < 0.001). Deck C was also selected more than Deck A (*p* < 0.001). Finally, a significant effect was found for Block 4 (when the relative risks and benefits of decisions should be very clear), F_(3, 822)_ = 55.694, *p* < 0.001, η^2^_*p*_ = 0.168. Participants selected more from Deck D than the remaining decks (*p*s < 0.009). Participants also selected more from Decks B and C than from Deck A (*p*s < 0.001). Thus, participants learned to decide more advantageously with additional trials by increasing the number of selections from a deck that maximized long-term rewards over a deck that provided more frequent immediate rewards (see Figure 2).

#### Delay discounting analyses

At the conclusion of the standard IGT (Block 2), no significant correlations were found between individual deck selections and delay discounting (*p*s > 0.086). A tendency to prefer more immediate (but smaller) rewards over larger (but more distant) rewards on the delay discounting task was associated with greater Deck B (*r* = 0.177, *p* = 0.017) but fewer Deck D (*r* = −0.189, *p* = 0.011) selections on Block 4. Thus, a preference for immediate gains on the IGT was also seen on a second decision making task.

#### Additional analyses

Additional analyses were conducted comparing the ratio of selections from Decks B and D for Blocks 2 and 4. For Block 2, the B/D ratio was 2.26 (*SD* = 4.54). For Block 4, the B/D ratio was 1.39 (*SD* = 1.83). A paired-samples *t*-test indicated that the preference for Deck B was greater for Block 2 than Block 4, *t*_(272)_ = 3.058, *p* = 0.002, providing further evidence of this shift toward consideration of long-term outcomes in deck decisions with additional learning trials.

We further analyzed whether the extent of delay discounting (*k*) could predict the B/D ratio for Block 2 and Block 4. For Block 2, the result was not significant, *F*_(1, 176)_ = 0.508, *p* = 0.477. However, for Block 4, performance on the delay discounting task was a significant predictor, *F*_(1, 176)_ = 4.289, *p* = 0.040, *R*^2^ = 0.024, *B* = 6.301. A preference for smaller, more immediate rewards on the delay discounting task predicted greater selections from Deck B than Deck D during Block 4.

#### Non-learners

Individual patterns of performance were also examined to determine what percentage of participants “failed to learn” on the first 100 trials (i.e., did not exhibit a preference for a deck). Seventy-three participants (26.5%) failed to develop a preference for a specific deck (or decks) on Trials 41–100, instead continuing to choose from each deck equally. Of those 73, 53 (75.7%) went on to develop a preference for a specific deck during the second 100 trials. Repeated measures ANOVAs indicated that this was due to increased Deck D selections, F_(1, 52)_ = 3.990, *p* = 0.051, η^2^_*p*_ = 0.071, and decreased Deck A selections, F_(1, 52)_ = 6.932, *p* = 0.011, η^2^_*p*_ = 0.118, from Block 2 to Block 4. Thus, with additional trials to learn from the feedback received, individuals who were classified as “non-learners” on the standard IGT eventually learned to choose advantageously on the task, and also showed a preference for a deck with positive long-term outcomes.

## General discussion

Individuals make numerous decisions on a daily basis that can have a tremendous impact on both current and future functioning. These decisions could include whether or not to keep smoking cigarettes, what car to buy or lease, whether to start a new relationship, or choosing between colleges that provide differing levels of monetary support. Many decisions are comprised of both short- and long-term gains and losses, and which of these components individuals choose to focus on will likely lead to different outcomes. Moreover, a myopic focus on the frequency of immediate rewards might result in the fallacious feeling that one has made a good decision, when in fact a focus on long-term consequences may have led to a better decision (Bechara et al., 1994).

In Study 1, deliberately inducing a negative mood altered decision making. Specifically, individuals in a negative mood began to choose more from Deck C, a deck associated with more frequent immediate losses but long-term positive outcomes, as the task progressed. This finding is in contrast to previous research that has shown a Deck B preference (e.g., Toplak et al., 2005; Caroselli et al., 2006; Fernie and Tunney, 2006). Past research has examined the relationship between decision making and self-reported (e.g., Kaplan et al., 2006; Suhr and Tsanadis, 2007; Smoski et al., 2008; Roiser et al., 2009a,b; Heilman et al., 2010) and manipulated (Harlé and Sanfey, 2007; de Vries et al., 2008) mood to determine the causal nature of mood's effects on decision making on the IGT, with mixed results. Moreover, extant research has examined the IGT by comparing preferences for Decks A and B (the disadvantageous decks based on long-term outcomes) to Decks C and D (the advantageous decks based on long-term outcomes), leading to an inability to examine discrepancies between the decks in terms of frequency of immediate rewards/losses and long-term outcomes. The present results indicate the importance of examining individual deck preferences on the IGT (e.g., Caroselli et al., 2006; Bechara, 2008; Buelow and Suhr, 2013; Buelow et al., 2013; Lin et al., 2013), as this process can shed light on these different components in the decision-making process.

The results of Study 2 indicated that decision making on the IGT can be improved with the administration of additional learning trials. Although at the start of the tasks participants selected significantly more from Decks B and D (i.e., the decks with losses on only 10% of trials), with additional trials in which to learn from feedback, they began to select from Deck D instead (the deck with a beneficial long-term outcome). This finding is consistent with the patterns of performance in several previous studies of decision making on the IGT, in which the pattern of selections showed an apparent “slowed learning curve” (e.g., Perretta et al., 2005; Delazer et al., 2009; Oyama et al., 2011). Although in many situations individuals may not be able to make repeated decisions, they can rehearse before making the decision, and increasing this practice may improve subsequent decisions. We also found that 76% of participants who failed to develop a deck preference on the standard IGT did so during the additional trials, and tended to be in favor of selecting more from Deck D and less from Deck A. Thus, providing additional learning trials to a large sample of control participants reversed a decision making impairment (if the standard 100-trial IGT was used to determine decision making deficits) as has been shown in clinical (Buelow et al., 2013) and smaller control samples (Lin et al., 2013). Future research should continue to investigate this tendency to develop a deck preference later in the task, as it is possible that personality characteristics and other factors can help predict who would benefit from additional learning trials on decision making tasks.

Additionally, the present results indicated that individuals who preferred more immediate rewards on the IGT also preferred more immediate rewards on a second decision making task. Specifically, participants who discounted future rewards on the delay discounting task made more selections from Deck B and fewer selections from Deck D. A participant who was better able to delay receipt of a reward in order to receive a larger reward showed advantageous decision making on the IGT, indicating that the participant was likely able to anticipate future outcomes (i.e., long-term gains) on both tasks (Petry et al., 1998). Our finding is consistent with other research showing individuals often make bad decisions due to discounting their future affective state (Kassam et al., 2008), or make more impatient decisions due to failure to anticipate future affect (Mitchell et al., 2011).

### Implications

The present studies are among the first to show that external manipulations can change decision making, and in some cases (Study 2), shift focus from the frequency of immediate gains/losses to long-term outcomes on the IGT. Previous research that manipulated long-term outcomes on the IGT to be even more positive has shown participants continue to focus on the frequency of gains/losses (Lin et al., 2007, 2009; Chiu et al., 2008), resulting in continued non-optimal decisions. In the present studies, we have shown that temporal focus can be shifted on this task, and that this shift is associated with performance on a second task. This shifting of focus has implications for the way in which the decision making process is conceptualized, as it could further differentiate types of decision making impairments. Administering a 200-trial version of the IGT, for example, could allow clinicians and researchers to determine if impairments seen on the task reflect difficulties learning from feedback or frank decision making impairment. This differentiation could lead to different personality and mood predictors, as well as potentially different outcomes on subsequent tasks.

In addition, the present study represents an advance from previous studies that focused on the original IGT scoring system (i.e., advantageous minus disadvantageous selections) that can mask deck-level preferences. The present results suggest that utilizing an individual deck-level approach can illuminate differences in focus on short- vs. long-term outcomes, or in the individual's weighting of the importance of these differing outcomes, and are in keeping with recent deck-level analysis trends (e.g., Buelow and Suhr, 2013; Steingroever et al., 2013). Administering a second measure of decision making that is sensitive to a focus on short- vs. long-term outcomes can also aid in the determination of whether disadvantageous decision making on the IGT is due to a myopic focus on immediate reward at the expense of positive long-term outcomes.

In addition, it may be worthwhile to investigate whether additional manipulations can be used to shift focus from immediate rewards to consideration of long-term outcomes. Finally, it would be important for future research to investigate whether the presence/absence of somatic markers during both early and later trials can predict who is more likely to decide advantageously after additional learning trials, as this may help to shed light on who learns and who does not learn with these additional practice trials.

Individuals who—in a standard single administration of the IGT—would have been classified as non-learners (either due to a lack of effort on the task or due to a failure to pick an advantageous strategy on the task)—were able to improve their decision making with additional trials. These data indicate that not all decision making deficits are equal, and that some individuals may simply need additional trials to learn from feedback during the decision making process. This difference is important for clinicians and researchers who utilize the IGT, as individuals who need more trials in which to learn to decide advantageously may reflect a different type of decision making impairment (i.e., less severe) than those who never learn to stay away from the bad decks. Thus, our data suggest that examining decision making using a binary outcome (i.e., bad/disadvantageous or good/advantageous) may not be sufficient on its own to detect more fine-grained decision making impairments. It is also important to understand what factors may be keeping a subgroup of participants from learning to decide advantageously, even after additional learning trials.

Finally, the present results suggest that both induced negative mood and additional learning trials can alter decision making on the IGT; however, it is unclear if these two factors may interact with one another to improve or impair decision making. For example, do individuals in a negative mood begin to select from Deck D even quicker during additional learning trials than someone in a positive mood? Or, does negative mood increase deliberative thinking and elaboration on decisions made during additional learning trials? Future research should investigate how these factors collectively affect decision making on the IGT and other tasks.

## Conclusions

The present studies sought to investigate two novel methods of improving decision making: inducing negative mood and providing additional learning trials. Inducing a negative mood altered decision making during the later trials, while providing additional learning trials improved decision making even among individuals who would have been classified as “non-learners” on the task. The current results also suggest the existence of subtle differences in temporal focus during the decision making process that should be an avenue for future exploration. Specifically, future research should investigate the implications of the present studies for decision making utilizing other behavioral and ecologically valid measures. Future research should also investigate the extent to which a focus on long-term outcomes vs. frequency of immediate rewards affects future attitude formation toward novel objects (e.g., Fazio et al., 2004), and how evaluation of affective valence alters decision making and risk-taking (e.g., Pietri et al., 2013).

### Conflict of interest statement

The authors declare that the research was conducted in the absence of any commercial or financial relationships that could be construed as a potential conflict of interest.

## Footnotes

^1^Item numbers from the IAPS for the learning trials: negative (1202, 1300, 2141, 2703, 2981, 3005.1, 3103, 3185, 3230, 6312, 6520, 6838, 7380, 9050, 9075, 9320, 9432, 9470, 9904, 9940), neutral (2036, 2107, 2191, 2359, 2374, 5390, 5410, 5731, 7019, 7021, 7026, 7033, 7041, 7052, 7057, 7061, 7081, 7175, 7500, 8312), and positive (1340, 1659, 1710, 1920, 2040, 2070, 2091, 2209, 2224, 2311, 2550, 4250, 4612, 5210, 5480, 7405, 7502, 8163, 8208, 8420).

^2^Additional IAPS pictures used during memory trials: negative (1019, 1230, 1275, 1280, 1302, 1321, 2095, 2220, 2980, 3022, 6230, 6570, 9163, 9630, 9911), neutral (1675, 2005, 2026, 2190, 2594, 5764, 5811, 7000, 7001, 7009, 7012, 7018, 7025, 7211, 7950), and positive (1500, 1540, 1595, 1604, 1660, 2035, 2151, 2540, 4574, 4575, 4597, 5202, 5814, 7325).
